# Expression of NLRP3 and AIM2 inflammasome in Peripheral blood in Chinese patients with acute and chronic brucellosis

**DOI:** 10.1038/s41598-022-19398-9

**Published:** 2022-09-06

**Authors:** Xiao Su, Shigang Zhao, Yijun Song

**Affiliations:** 1 Tianjin Medical University, Tianjin, China; 2 Department of Neurology, Affiliated Hospital of Inner Mongolia Medical University, Hohhot, China; 3 General Medicine Department, Tianjin Medical University General Hospital, Tianjin, China; 4 Department of Neurology, Tianjin Medical University General Hospital, Tianjin, China; 5 Key Laboratory of Post-Neuroinjury Neuro-repair and Regeneration in Central Nervous System, Ministry of Education and Tianjin Neurological Institute, Tianjin, China

**Keywords:** Diseases, Infectious diseases, Bacterial infection

## Abstract

Brucellosis is a zoonotic disease caused by *Brucella abortus*. An efficient immune response is crucial for curing brucellosis. The inflammasome plays a significant role in the immune response. It is unclear which inflammasome is active in acute and chronic brucellosis and how its levels relate to inflammatory cytokines. A total of 40 patients with acute or chronic brucellosis and 20 healthy volunteers had peripheral blood samples collected. The expression levels of AIM2, NLRP3, ASC, and Caspase-1 were determined by a real-time polymerase chain reaction from RNA and serum samples, and IL-1β, IL-18, and IFN-γ were measured by enzyme-linked immunosorbent assay. In the acute brucellosis group, AIM2 expression was significantly higher, while ACS expression was significantly lower than that of healthy volunteers. In patients with chronic brucellosis, AIM2 expression was significantly lower, while Caspase-1 expression was significantly higher than that of healthy volunteers. Serum IL-18 and IFN-γ levels were significantly higher in patients with acute brucellosis than in healthy controls. The IFN-γ level was also significantly higher in patients with chronic brucellosis than in healthy controls. The inflammasome responds differently in different stages of brucellosis. The inflammasome may be the site of action of immune escape in brucellosis.

## Introduction

Brucellosis is the most prevalent bacterial zoonosis worldwide^1^. In recent years, human brucellosis incidence has increased sharply. The overall incidence of human brucellosis in mainland China increased from 0.92 cases/100,000 in 2004 to 4.2 cases/100,000 in 2014^2–4^. Brucellosis has a wide spectrum of clinical manifestations, which may last for days or even years, lacking typical clinical manifestations; moreover, it is often misdiagnosed, which causes inadequate therapy and prolonged disease^5^. The clinical forms of human brucellosis are typically classified as acute (< 3 months) and chronic (> 1 year), depending on the duration of symptoms. The immune response varies according to clinical forms^6,7^.

Once Brucella invades the mucosa, professional phagocytes located in the submucosa will ingest the bacteria through zipper-like phagocytosis^8^. Brucella is incorporated into macrophages and monocytes through the action of complement and Fc receptors Brucella survives and replicates within phagocytes, evades and modulates the host immune response, and spreads to preferred tissues with the blood^9,10^. Phagocytes delivery of bacterial peptides associated with MHC-I and II to T lymphocytes is performed^6^. The T cell is activated by recognizing peptide-MHC complexes through receptors on their surface. The T cells differentiate from naive T cells into T helper type 1 (Th1) cells under the action of IL-12 secreted by antigen-presenting cells^11,12^. Although Brucella is readily internalized by neutrophils^8,13^ in the early stages of disease, neutrophils are rarely activated^14,15^. Inhibition of caspase-1 did not hinder BrLPS-induced PMN cell death, suggesting that the inflammasome pathway is not involved after Brucella invasion of neutrophils^16^. In acute brucellosis, T helper-1 (Th1) cytokines, mainly interferon-gamma (IFN-γ) and interleukin (IL)-2, are overproduced in the intracytoplasmic niche and serum^17^. The IL-2 and IFN-γ produced by Th1 cells are essential for the clearance of Brucella. Any disturbance of the Th1 response may contribute to the development of chronic brucellosis and poor prognosis. However, in patients with chronic brucellosis, low CD3 + IFN-γ+ levels suggest a malfunctioning Th1 response^18^. In the chronic phase of the disease, Brucella mainly survives and replicates for a long time in mononuclear macrophages^19^. The mechanism of incomplete activation of the immune response in brucellosis has been a hot research topic. Macrophages sense pathogens mainly through pattern recognition receptors, control pathogen infection, and the production of cytokines and antimicrobial intermediates^20^. In recent years, many studies have identified that intracellular recognition receptors formed by inflammasomes play a key role in controlling Brucella infection. Macrophages recognize Brucella through pattern recognition receptors (PRRs) such as NLRs (Nod-like receptors), which activate the inflammasome, triggering the production of pro-inflammatory cytokines, leading to the development of a subsequent pattern of type 1 immune responses, which is critical for Brucella clearance and infection control^21^. Inflammasomes are crucial not only in eliminating the pathogen by inducing cysteine-dependent aspartate specific protease-1 (Caspase-1)-associated pyroptosis but also in inducing the adaptive cellular immune response by inducing the secretion of pro-inflammatory cytokines IL-1β and IL-18^22^.

Inflammasomes are complexes composed of multiple proteins involved in pattern recognition receptors. The complexes consists of receptor proteins, the apoptosis-associated speck-like protein (ACS) containing a caspase recruitment domain (CARD) and Caspase-1^23^. Receptor proteins include the NLR family (Nod-like receptors, NLRs) and the HIN200 family (interferon-inducible p200-protein). The NLR family and the HIN200 families are represented by the NLRP3 inflammatory factor and the Absent in Melanoma 2 (AIM2) receptor protein, respectively^24^. ASC is a protein-activating molecule associated with apoptosis, which is composed of a pyrin domain (PYD) at the N-terminus and a CARD at the C-terminus. Additionally, ASC can promote apoptosis directly or by activating downstream caspases^25^.

After the invasion of pathogenic microorganisms, AIM2 or NLRP3 can recruit Caspase-1 precursor (pro-Caspase-1) through ASC to activate Caspase-1. Active Caspase-1 subsequently functions to cleave the pro-inflammatory IL-1 family of cytokines into their bioactive forms, IL-1β and IL-18, and induce pyroptosis, an inflammatory cell death^26^. Through in vitro experiments, Marinu et al. confirmed that *B. abortus* ligands activate NLRP3 and AIM2 inflammasomes and control infection through the inflammatory response caused by IL-1β and IL-18^27^. Miraglia et al. confirmed that infected astrocytes and microglia from C57BL/6, NLRP3, andAIM2 KO mice with *B. abortus*. IL-1b secretion induced by *B. abortus* infection was completely abolished in AIM2 and NLRP3 astrocytes and microglia when compared with WT glial cells. These results demonstrate that in glial cells infected with *B. abortus*, the activation of ASC-dependent inflammasomes and the production of IL-1b are dependent on NLRP3 and AIM2^28,29^.

Most current studies on the NLRP3 and AIM2 inflammasomes, are conducted on animals, whose immune mechanisms differ from those of humans. Inflammasome activation and inflammatory cytokine levels in acute and chronic brucellosis have not been undetected. By analyzing the transcriptional expression of peripheral blood caspase-1 and associated inflammasomes in patients with acute brucellosis, Karaca et al. found that in acute brucellosis, caspase-1 and associated inflammasomes are activated, thereby inducing the secretion of cytokines, such as IFN-γ and IL-18, which continue to induce a cellular immune response^30^. However, the main danger of Brucella is that it easily escapes clearance by the immune system, leading to the development of chronic brucellosis, which imposes a huge burden on patients. Therefore, studying the changes of the inflammasome in patients with brucellosis in different periods may provide clues for revealing the immune escape mechanism of brucellosis.

The present study aims to investigate the expression levels of inflammasomes, such as AIM2 and NLR family PYD-domain-containing 3 (NLRP3), in peripheral blood samples from patients with acute or chronic brucellosis and the levels of inflammatory cytokines (such as IL-1β, IL-18, and IFN-γ) in the systemic circulation.

## Materials and methods

This study conforms to the ethical guidelines of the Declaration of Helsinki and has been approved by the Medical Ethics Committee of the Affiliated Hospital of Inner Mongolia Medical University (batch number: YKD202106066). All patients signed a written informed consent before participating in our study. A total of 40 patients, 20 with acute brucellosis and 20 with chronic brucellosis, were enrolled in the study.

Refer to "Diagnosis of Brucellosis" (People’s Republic of China Health Industry Standard WS269-2019).

Acute brucellosis is defined as persistent symptoms of brucellosis for < 3 months with one of the following conditions: Rose Bengal plate agglutination test (>++); serum agglutination test (≥ 1:100++).

Chronic brucellosis refers to persistent symptoms of brucellosis for > 1 year with one of the following conditions: Rose Bengal plate agglutination test (>++); serum agglutination test (≥ 1:50++ or ≥ 1:100++).

All patients were collected from the Inner Mongolia Center for Disease Control and Prevention. Staff from the Inner Mongolia Center for Disease Control and Prevention asked all patients for their detailed medical histories which include epidemiological history, clinical symptoms, duration of clinical symptoms and antibiotics use. We included patients with newly diagnosed brucellosis who were not treated with antibiotics to avoid the possible effects of antibiotics on the immune system. None of the patients received antibiotics (see Supplementary material Table S1 online).

The control group consisted of 20 age-matched healthy volunteers who were negative for brucellosis based on serological tests and clinical data. All controls were free of common infectious, chronic or autoimmune diseases.

### Real-time polymerase chain reaction (RT-PCR) analysis

Patients with acute and chronic brucellosis as well as healthy controls were collected for peripheral blood sampling (empty stomach in the morning).

Total RNA samples were extracted from peripheral blood using RNAiso Blood (Takara, japan) according to the manufacturer’s instructions.

The quality and quantity of RNA samples were evaluated by MaestroNano Spectrophotometer, and integrity was confirmed by electrophoresis (1.5% denaturing agarose gel electrophoresis) (see Supplementary Fig S1 online).

In addition, 1 μg of RNA for cDNA synthesis was reverse-transcribed in a T100 Thermal Cycler PCR instrument using the PrimeScript RT reagent Kit with gDNA Eraser (Perfect Real Time) according to the manufacturer’s instructions.

For RT-qPCR reactions, cDNA was mixed with B Green Premix Ex Taq II (Tli RNaseH Plus) and then aliquoted into the tubes containing primers. The primers, synthesized by Shanghai Sangong Company, were designed using Primer5 software according to the cDNA or mRNA sequence information of human NLRP3, AIM2, ASC, Caspase-1, and internal reference gene Beta-actin published on NCBI. Primer sequences are listed in Table 1. The standard curve of qPCR amplification efficiency is shown in Supplementary Materials Table S2 and Figure S2.

**Table 1:** Primer sequence listing.

Primer name	base Sequence
NLRP3-F	5′-AGCCCCGTGAGTCCCATTA-3′
NLRP3-R	5′-ACGCCCAGTCCAACATCATCT-3′
AIM2-F	5′-ATCTCCTGCTTGCCTTCTTGG-3′
AIM2-R	5′-AAGTCTCTCCTCATGTTAAGCCTG-3′
ASC-F	5′-AGTGGCTGCTGGATGCTCTG-3′
ASC-R	5′-CATCTTGCTTGGGTTGGTGG-3′
Caspase-1-F	5′-TGAATACCAAGAACTGCCCAAG-3′
Caspase-1-R	5′-GCATCATCCTCAAACTCTTCTGTAG-3′
Beta-actin-F	5′-CCTGGCACCCAGCACAAT-3′
Beta-actin-R	5′-GGGCCGGACTCGTCATAC-3′

The final volume for reactions was 20 ul, including 10 ul of TB Green Premix Ex Taq II (Tli RNaseH Plus), 0.8 ul of PCR Forward Primer (10 μM), 0.8 ul of PCR Forward Primer (10 μM), 0.4 ul of ROX Reference Dye or Dye II, and 2 ul of cDNA solution.

**Figure 1:**
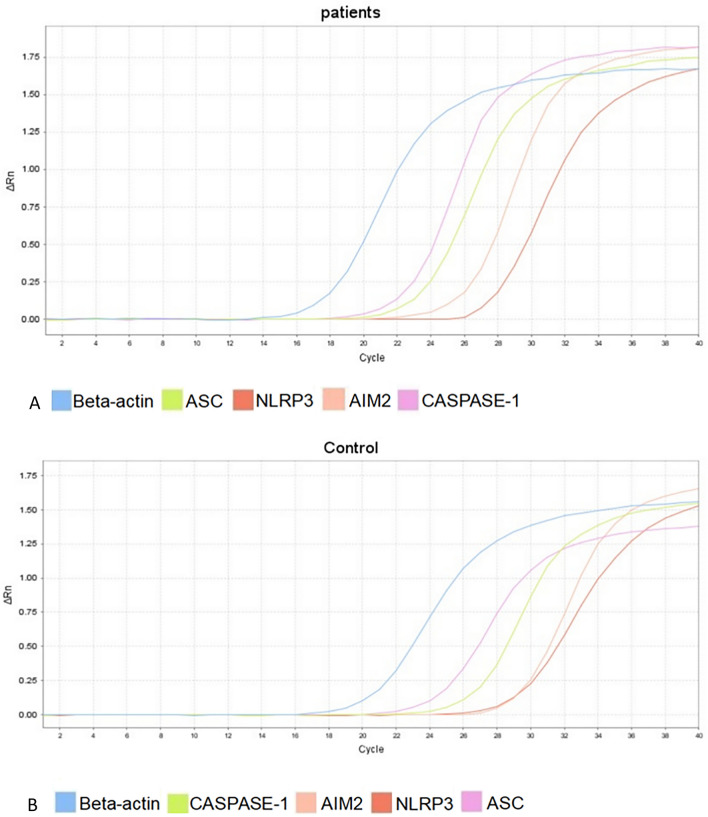
Representative amplification curves (**A**) Representative amplification curves of cDNA from patient groups for each gene. (**B**) Representative amplification curves of cDNA from control groups for each gene. Total RNA samples were extracted from peripheral blood. 1 μg of RNA was used for reverse transcription synthesis of cDNA. RT-PCR was subsequently performed with cDNA.

Three technical replicates were performed for each gene in each sample. Genes were mixed with gentle shaking and centrifuged briefly. Additionally, PCR conditions were repeated for 40 cycles according to the manufacturer’s instructions. Real-time PCR reactions were performed using the Applied Biosystems 7500 Fast Real-Time PCR System. The amplification and melting curves of real-time PCR were confirmed after completing the reaction (Fig. 1A,B).

The CT value was automatically generated by the fluorescence quantitative PCR instrument analysis software Applied Biosystems 7500 Fast Real-Time PCR System. The relative transcription levels of NLRP3 and AIM2 inflammasome-related genes were calculated by the 2−ΔΔct method^31^ and 2−ΔΔct = 2 − [(the target gene CT in the experimental group − the internal reference gene CT in the experimental group) − (the target gene CT in the control group − the internal reference gene CT in the control group)]. According to the results, a bar chart was drawn to indicate the relative expressions and changing trends of NLRP3, AIM2, ASC, and Caspase-1.

### Cytokine detection

All controls and serum samples from brucellosis patients were separated from their peripheral blood and kept at − 80 °C until the study. Serum samples were tested for IL-1β, IL-18, and IFN-γ levels using commercially available ELISA kits (BOSTER, China) according to the manufacturer’s instructions.

### Statistical analysis

Statistical analysis was performed using SPSS 18.0 (SPSS Inc., Chicago, USA) software, and data were graphically represented using GraphPad Prism 8.0 software. The measurement data were described by (X ± SD) or P50 (P25, P75). The normality test was performed by the single-sample KS goodness-of-fit method. Means of multiple groups were compared for normal distribution through the one-way ANOVA test. For data that does not obey the normal distribution, the Kruskal–Wallis rank-sum test and the Mann–Whitney rank-sum test were used to compare multiple groups and two groups, respectively. The count data were described by the rate, and statistical inference was performed via the X^2^ test. Spearman correlation analysis was used to explore the correlation between inflammasome gene expression and cytokines. *P* < 0.05 difference was statistically significant.

### Ethics declarations

This study was performed in line with the principles of the Declaration of Helsinki. Approval was granted by the Ethics Committee of the Affiliated Hospital of Inner Mongolia Medical University (Date2021.3.3/NoYKD202101066). Informed consent was obtained from all individual participants included in the study.

## Results

There is no significant difference in age and gender of the included patients (*P* > 0.05). Table 2 shows the results of the tiger red plate test and the standard agglutination method of the subjects. The duration of acute brucellosis ranges from 3 days to 2 months, with an average duration of 23 days; the duration of chronic brucellosis varies from 1 year to more than 10 years, with half of the cases lasting 1–2 years and an average duration of 3 years. In this study, the most common clinical symptoms in patients with acute brucellosis were fever (55%), followed by sweating (35%), arthralgia (30%), muscle pain (25%), fatigue (25%), headache (5%), cardiac (5%) and orchitis (5%). The most common clinical symptom in patients with chronic brucellosis is arthralgia (60%), followed by fatigue (35%), sweating (35%), muscle pain (35%), fever (30%), and headache (10%).

**Table 2:** Baseline table of case group and healthy group.

Stage	Acute brucellosis	Chronic brucellosis	Healthy control
Age	40.65 ± 14.911	44.45 ± 12.898	38.00 ± 10.973
**Gender**			
Man	15	12	13
Woman	5	8	7
IGG	38.49 ± 36.1	3.57 ± 2.81	1.89 ± 1.69
IGM	13.75 ± 16.13	39.1 ± 28.41	5.31 ± 3.28
RBPT	++	++	0
**SAT**			
1:50++	0 (0)	8 (40)	0
1:100++	10 (50)	7 (35)	0
1:100+++	1 (5)	3 (15)	0
1:100++++	9 (45)	2 (10)	0
**Clinical syndromes (%)**			
Fever	11 (55%)	6 (30%)	0
Fatigue	5 (25%)	7 (35%)	0
Sweats	7 (35%)	7 (35%)	0
Muscle pain	5 (25%)	7 (35%)	0
Headache	1 (5%)	2 (10%)	0
Arthralgia	6 (30%)	12 (60%)	0
Cardiac	1 (5%)	0 (0%)	0
Orchitis/epididymitis	1 (5%)	0 (0%)	0

### Inflammasome genes expression levels

**Figure 2:**
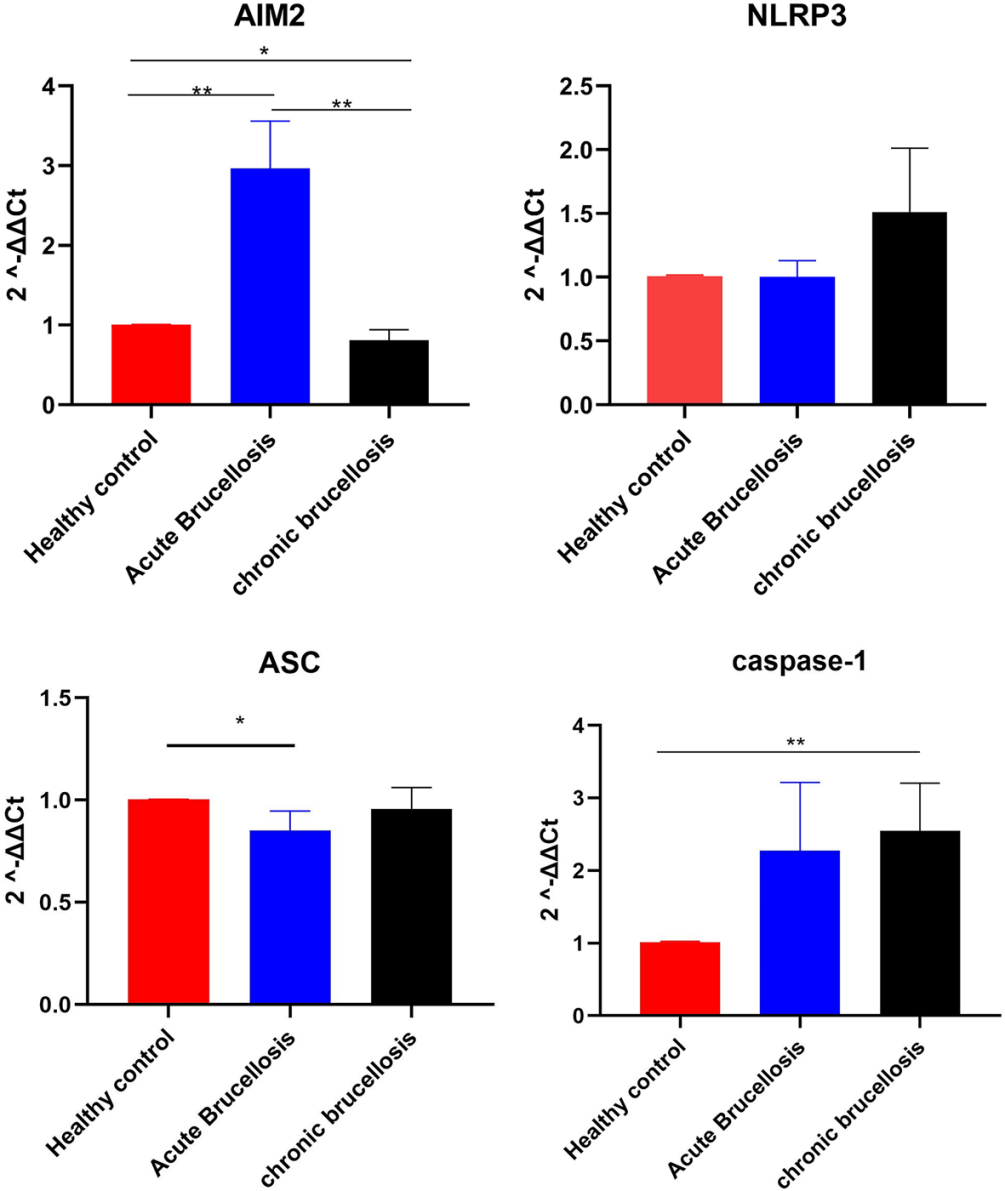
Description and differences of NLRP3, AIM2, ASC, Caspase-1 gene expression levels in each experimental group. The CT value was automatically generated by the fluorescence quantitative PCR instrument analysis software. The gene relative transcription levels were calculated by the 2−ΔΔct method. Analysis of the relative transcript levels of AIM2, NLRP3, ASC and caspase-1 genes in healthy control group, acute brucellosis group and chronic brucellosis group. Beta-actin has been used as a housekeeping gene. *symbolizes statistically significant change in the acute or chronic brucellosis group in comparison with healthy control. 2−ΔΔct were presented with ± SE. **P < 0.01,*P < 0.05.

The expression levels of AIM2, NLRP3, ASC, and Caspase-1 were investigated in patients with acute brucellosis and chronic brucellosis as well as healthy controls. Compared with healthy controls, patients with acute brucellosis show significantly higher AIM2 expression and significantly lower ACS expression, with no significant changes in NLRP3 but an increasing tendency of Caspase-1 genes. Compared with healthy controls, chronic brucellosis patients present an increasing tendency of NLRP3, significantly decreased AIM2 expression, and significantly increased Caspase-1 expression, with no significant changes in ASC (Fig. 2 and Table 3).

**Table 3:** Description and differences of gene expression levels (2−ΔΔct) and inflammatory cytokines (pg/ml) in each experimental group *P < 0.05 **P < 0.01.

Stage	Acute	Chronic	Control	Χ^2^
NLRP3	0.78 (0.63, 1.37)	0.71 (0.39, 1.77)	1.00 (1.00, 1.00)	0.893
AIM2	2.17 (1.15, 3.87)	0.63 (0.29, 1.46)	1.00 (1.00, 1.01	19.595**
ASC	0.85 (0.53, 0.96)	0.91 (0.56, 1.28)	1.00 (1.00, 1.00)	8.086*
CAPASE-1	1.24 (0.74, 1.52)	1.53 (1.07, 2.47)	1.00 (1.00, 1.01)	9.302*
IL-18	2665.81 (1446.15, 6038.79)	1092.63 (794.23, 1619.82)	1333.25 (1063.72, 1889.29)	15.860*
IL-1β	0.23 (0.00, 7.04)	0.00 (0.00, 1.49)	0.63 (0.00, 2.50)	0.788
IFN-γ	4.38 (0.12, 17.35)	0.14 (0.00, 1.18)	0.00 (0.00, 0.00)	19.653*

### IL-1β, IL-18, and IFN-γ levels

**Figure 3:**
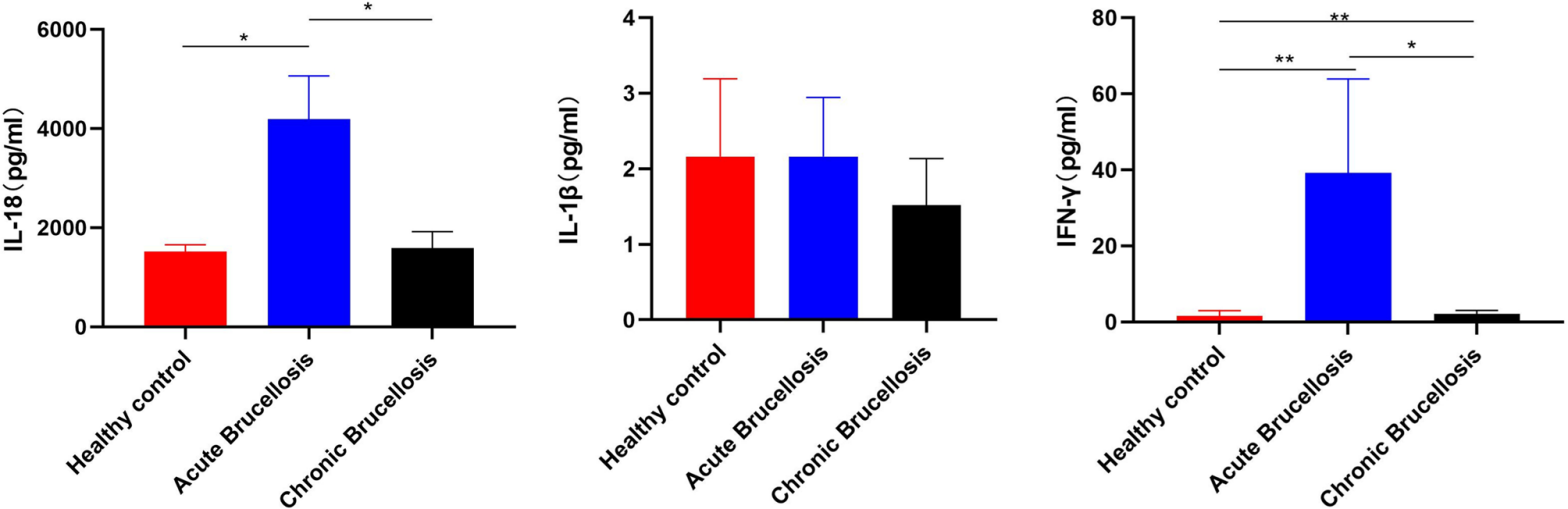
The description and differences of the expression levels (pg/ml) of inflammatory cytokines IL-18, IL-1β and IFN-γ in each experimental group. Serum IL-1β, IL-18, and IFN-γ mean values in healthy control, acute brucellosis and chronic brucellosis volunteers. Mean ± SD were presented in each group. *symbolize statistical differences of IL-18 and IFN-γ values between the healthy group and brucellosis group, respectively **P < 0.01,*P < 0.05.

The levels of IFN-γ, IL-1β, and IL-18 were investigated in serum samples from each patient and healthy controls. IFN-γ, a cytokine representing the cellular immune response, is significantly increased in both acute and chronic brucellosis patients. For the cytokines IL-1β and IL-18 secreted after pro-Caspase-1 activation, IL-18 is significantly increased in patients with acute brucellosis, while IL-1β shows an increasing trend but is not statistically significant; in chronic brucellosis patients, IL-18 and IL-1β levels are not statistically different from healthy controls (Fig. 3).

The correlation between cytokines and inflammasome gene expression was analyzed. The expression of the AIM2 receptor gene in patients with acute brucellosis is positively correlated with IL-18 expression (r = 0.591 and *P* = 0.006). The expression of the NLRP3 receptor gene in patients with acute brucellosis negatively correlates with IL-18 expression (r = − 0.454 and *P* = 0.044). The expression of the AIM2 receptor gene in patients with chronic brucellosis is positively correlated with IFN-γ expression (r = 0.456 and *P* = 0.044). The expression of ASC and Caspase-1 is not correlated with cytokine levels (*P* < 0.05) (Tables 4 and 5).

**Table 4:** Acute brucellosis correlation analysis of inflammasome and inflammatory cytokines.

Variable	r/P	IL-18	IL-1β	IFN-γ	Caspase-1	ASC	AIM2	NLRP3
AIM2	r	0.591	0.194	0.417	0.039	0.394	1	− 0.086
	P	0.006**	0.413	0.068	0.870	0.086		0.719
NLRP3	r	− 0.454	0.199	− 0.229	− 0.024	0.392		1
	p	0.044*	0.401	0.331	0.920	0.087		
Caspase-1	r	− 0.108	− 0.312	− 0.301	1	0.317	0.039	− 0.024
	P	0.650	0.181	0.197		0.173	0.870	0.920
ASC	r	0.198	0.150	− 0.021	0.317	1	0.039	− 0.392
	p	0.402	0.527	0.930	0.173		0.870	0.087
IL-18	r	1	0.042	0.456	− 0.108	0.198	0.591	− 0.454
	P		0.861	0.043	0.65	0.402	0.006	0.044
IL-1β	r	0.042	1	0.361	− 0.312	0.150	− 0.194	0.199
	p	0.861		0.118	0.181	0.527	0.413	0.401
IFN-γ	r	0.456	0.361	1	− 0.301	− 0.021	0.417	− 0.229
	P	0.043*	0.118		0.197	0.930	0.068	0.331

*P < 0.05 **P < 0.01.

**Table5:** Chronic Brucellosis Correlation analysis of inflammasome and inflammatory cytokines.

Variable	r/P	IL-18	IL-1β	IFN-γ	Caspase-1	ASC	AIM2	NLRP3
AIM2	r	− 0.358	0.194	0.456	− 0.298	0.408	1	0.429
	P	0.121	0.413	0.044*	0.202	0.075		0.059
NLRP3	r	− 0.436	0.199	0.227	− 0.420	0.414	0.429	1
	p	0.055	0.401	0.336	0.066	0.07	0.059	
Caspase-1	r	− 0.101	− 0.312	− 0.233	1	0.027	− 0.298	− 0.420
	P	0.673	0.181	0.322		0.910	0.202	0.066
ASC	r	− 0.358	0.15	0.176	0.027	1	0.408	0.414
	p	0.121	0.527	0.459	0.910		0.075	0.07
IL-18	r	1	0.042	0.311	− 0.101	− 0.358	0.029	− 0.436
	P		0.861	0.182	0.673	0.121	0.905	0.055
IL-1β	r	0.042	1	0.361	− 0.312	0.15	0.194	0.199
	p	0.861		0.118	0.181	0.527	0.413	0.401
IFN-γ	r	0.311	0.361	1	− 0.233	0.176	0.456	0.227
	P	0.182	0.118		0.322	0.459	0.044	0.336

*P < 0.05.

## Discussion

This study aims to investigate the expression levels of AIM2, NLRP3, ASC, and Caspase-1 inflammasomes in acute and chronic brucellosis patients to reveal the possible mechanism of Brucella immune escape. The main conclusion is that the inflammasome is differentially expressed in patients with brucellosis at different stages. The inflammasome may be the site of action of immune escape in brucellosis.

Brucellosis is one of the most common zoonotic infectious diseases worldwide and is caused by the intracellular pathogen Brucella. Brucella can survive and replicate in host cells, evading the host immune response by expressing different virulence factors and adopting various escape strategies, which leads to the evolution of a part of the disease from acute to chronic. Whether the innate and acquired immune responses are sufficiently active after the onset of human brucellosis is still under discussion^32,33^.

The Brucella can release molecules such as its own DNA, which subsequently induces the production and release of pro-inflammatory cytokines in the host immune system, as detected by cytosolic AIM2 inflammasomes. Gomes et al. support the idea that Brucella genomic DNA is a ligand for AIM2 inflammasomes. In addition, AIM2 knockout mice are more susceptible to Brucella infection and infection of C57BL/6, NLRC4, NLRP3, AIM2, ASC and Caspase-1 KO (knockout) BMDM (bone marrow-derived macrophage) with *B. abortus* than wild-type control mice^27^. The BMDM-induced IL-1β secretion is completely abolished in ASC and Caspase-1 KO macrophages and significantly reduced in AIM2 and NLRP3 KO cells. The results suggest that ASC-dependent inflammasome activation and IL-1β production partially depend on NLRP3 and AIM2^34^. However, AIM2 inflammasome changes in patients with brucellosis at different stages remain unknown. In this study, AIM2 expression is significantly higher in patients with acute brucellosis but lower in chronic brucellosis patients than in healthy controls. Additionally, AIM2 positively correlates with IL-18 expression in acute brucellosis patients and IFN-γ expression in chronic brucellosis patients. The results suggest that AIM2 may play a vital role in activating adaptive immune responses in brucellosis patients. We speculate that activation of ASC-dependent inflammasomes in human brucellosis is more dependent on the AIM2 receptor recognition of Brucella DNA than the activation of the NLRP3 receptor. Moreover, we found differences in AIM2 between acute and chronic brucellosis. The expression of AIM2 is higher in acute brucellosis patients but lower in chronic brucellosis than in healthy controls. In the early stage of Brucella infection in humans, the immune system of the host can timely recognize Brucella through the AIM2 receptor and initiate a cascade reaction to control the infection by producing inflammatory factors. However, as the disease progresses, Brucella may inhibit or down-regulate AIM2 expression to evade clearance by the host immune system.

Studies have shown that NLRP3 knockout mice have lower levels of IL-1β secretion in macrophages and are more susceptible to Brucella infection than control mice^27^. Chen et al. demonstrated that most transcription of NLRP3 inflammasome-related genes was transiently inhibited in the first 24 h of Brucella infection of mouse macrophages RAW264.7. Additionally, IL-1β and IL-18 in the corresponding cell supernatant content were reduced^35^. In this study, no significant difference was found in NLRP3 expression between acute brucellosis, chronic brucellosis, and healthy control; there was no statistical significance in expression between each group. The result is basically consistent with the speculation of Chen et al. By combining with the trend changes, we can speculate that the sensitivity of early NLRP3 to Brucella antigens is not high and is in a stage of low response or even inhibition in the initial disease phase, consistent with the characteristics of Brucella escaping host immune attack and causing chronic infection.

Tupik et al. performed in vivo studies using Asc −/− mice and observed decreased mouse survival, decreased immune cell recruitment, and increased bacterial load^36^. Evidence has shown that ASC is essential in the Caspase-1 response to *Brucella abortus*. In our study, ASC expression is lower in acute brucellosis patients than in healthy controls but is not different in chronic brucellosis patients. Therefore, we speculate that Brucella may inhibit or down-regulate the expression of the important protein ASC in the inflammasome at the early invasion stage into the human body, thereby inhibiting strong inflammatory responses and avoiding its elimination by the host immune system. This way may allow Brucella to escape and eliminate the immune system. Then, as the disease progresses, the host immune response gradually increases and expression of ASC tends to increase.

Inflammasomes are large multimolecular complexes best known for their ability to control activation of the proteolytic enzyme caspase-1^37^. Caspase-1 in turn regulates the proteolytic maturation of interleukin-1b and IL-18, as well as rapid, noxious, inflammatory form of cell death termed pyroptosis^38^. During the acute phase of brucellosis infection in mice, Gomes et al. infected C57BL/6 and NLRC4, NLRP3, AIM2, ASC and caspase-1 KOBMDMs with Brucella. Brucella infection induced IL-1β secretion in ASC and caspase-1 completely disappeared in KO macrophages. These results suggest that caspase-1 is essential for the secretion of IL-1β in the acute phase of Brucella infection. During the chronic phase of murine brucellosis, mice lacking the inflammasome component caspase-1 were more susceptible to brucellosis infection. These mice showed higher bacterial counts in the spleen at 4 weeks post-infection, which was partly attributable to a reduction in IL-1β and possibly IL-18 production and/or other caspase-1-dependent processes^27^. Active Caspase-1 allows the host to control various microbial infections. Additionally, Caspase-1 activation induced by *Brucella abortus* does not cause macrophage scorch death^27^. The expression level of Caspase-1 shows no significant increase in acute brucellosis and no correlation with IFN-γ and IL-18. Moreover, its expression may reduce IL-1β expression in patients with acute brucellosis, which may be a strategy for Brucella not to lose its niche. The gene expression of Caspase-1 is higher in the chronic phase than in the healthy control group. We speculate that Caspase-1 may play a major role in chronic brucellosis, which may relate to the overall gradual enhancement of the host immune response as the disease progresses.

Except for the two representative inflammasomes, NLRP3 and AIM2. NLRP1, NLRC4, and IFI16 can also form multi-protein complex inflammasomes that induce caspase-1 activation by acting as sensors of PAMPs or risk-associated molecular patterns. The NLRP1 inflammasome in mice can be activated by the lethal toxin of Bacillus anthracis^39^, but NLRP1 gene transcription was not detected in Brucella-infected macrophages^40^. The NLR apoptosis inhibitory protein (NAIP)/NLRC4 inflammasome directly recognizes bacterial flagellin and type III secretion system components^41^. However, NLRC4 is not involved in sensing inflammasome activation following Brucella infection which showed that NLRC4 is not an indispensable receptor during caspase-1 activation and IL-1βsecretion following Brucella infection. IFI16, can also bind DNA and engage ASC, leading to inflammasome activation^42^. The remaining NLR receptors do not form inflammasomes but play other roles in the immune system. NLRP12 regulates NF-kB, IL1-β and Caspase-1. Tatiana et al. Show that NLRP12 plays an important role in negatively regulating the early inflammatory response against *Bacillus abortus*^43^. Studying all these relevant receptors may provide new insights into the mechanisms of immune escape following Brucella infection.

## Conclusions

In conclusion, inflammasome is differentially expressed in patients with brucellosis at different stages. Cytokines (IL-18, IFN-γ) associated with inflammasome changes are also altered at different stages of brucellosis. The changes may relate to the immune escape of Brucella. Given the paucity of studies on inflammasome in human brucellosis specimens, especially in chronic brucellosis, this study may suggest the mechanism of the immune escape of Brucella in humans and positively affect future vaccine production and disease prevention.

### Limitation

This study is limited to one province in mainland China with a small sample size. Future research needs to expand the study area and increase the number of subjects, and increase the amount of blood samples to study protein expression to obtain more sufficient evidence to support.

## Supplementary Information

Supplementary Information.

## Data Availability

The datasets generated during and/or analysed during the current study are not publicly available due to [involving privacy] but are available from the corresponding author on reasonable request.

## References

[1] Franco MP, Mulder M, Gilman RH, Smits HL (2007). Human brucellosis. Lancet Infect. Dis..

[2] Zhang J, Yin F, Zhang T, Yang C, Zhang X, Feng Z, Li X (2014). Spatial analysis on human brucellosis incidence in mainland China: 2004–2010. BMJ Open.

[3] Lai S (2017). Changing epidemiology of human brucellosis, China, 1955–2014. Emerg. Infect. Dis..

[4] Li YJ, Li XL, Liang S, Fang LQ, Cao WC (2013). Epidemiological features and risk factors associated with the spatial and temporal distribution of human brucellosis in China. BMC Infect. Dis..

[5] Ramin B, Macpherson P (2010). Human brucellosis. BMJ.

[6] Skendros P, Boura P (2013). Immunity to brucellosis. Rev. Sci. Tech..

[7] Skendros P, Pappas G, Boura P (2011). Cell-mediated immunity in human brucellosis. Microbes Infect..

[8] Ackermann MR, Cheville NF, Deyoe BL (1988). Bovine ileal dome lymphoepithelial cells: endocytosis and transport of *Brucella*
*abortus* strain. Vet. Pathol..

[9] Carvalho Neta AV (2008). Modulation of the bovine trophoblastic innate immune response by *Brucella*
*abortus*. Infect. Immun..

[10] Delpino MV, Fossati CA, Baldi PC (2009). Proinflammatory response of human osteoblastic cell lines and osteoblast-monocyte interaction upon infection with *Brucella* spp. Infect. Immnu..

[11] Manetti R, Parronchi P, Giudizi MG, Piccinni MP, Maggi E, Trinchieri G, Romagnani S (1993). Natural killer cell stimulatory factor (interleukin 12 [IL-12]) induces T helper type 1 (Th1)-specific immune responses and inhibits the development of IL-4-producing Th cells. J. Exp. Med..

[12] Saito S, Sakai M, Sasaki Y, Tanebe K, Tsuda H, Michimata T (1999). Quantitative analysis of peripheral blood Th0, Th1, Th2 and the Th1:Th2 cell ratio during normal human pregnancy and preeclampsia. Clin. Exp. Immunol..

[13] Braude AI (1951). Studies in the pathology and pathogenesis of experimental brucellosis. II. The formation of the hepatic granuloma and its evolution. J. Infect. Dis..

[14] Martínez de Tejada G, Pizarro-Cerdá J, Moreno E, Moriyón I (1995). The outer membranes of *Brucella* spp. are resistant to bactericidal cationic peptides. Infect. Immun..

[15] Kreutzer DL, Dreyfus LA, Robertson DC (1979). Interaction of polymorphonuclear leukocytes with smooth and rough strains of *Brucella*
*abortus*. Infect. Immun..

[16] Barquero-Calvo E (2015). *Brucella*
*abortus* Induces the Premature Death of Human Neutrophils through the Action of Its Lipopolysaccharide. PLoS Pathog..

[17] Baldwin CL, Goenka R (2006). Host immune responses to the intracellular bacteria *Brucella*: Does the bacteria instruct the host to facilitate chronic infection. Crit. Rev. Immunol..

[18] Kayhan, B., Kayabas, U., Kolgelier, S., Otlu, B., Gul, M., Kurtoglu, E. L. & Bayindir, Y. Mystery of immune response in relapsed Brucellosis: immunophenotyping and multiple cytokine analysis (2016).

[19] Atluri VL, Xavier MN, de Jong MF, den Hartigh AB, Tsolis RM (2011). Interactions of the human pathogenic *Brucella* species with their hosts. Annu. Rev. Microbiol..

[20] Hedayatizadeh-Omran A, Rafiei A, Hajilooi M, Haghshenas M (2010). Interferon-gamma low producer genotype +5644 over presented in patients with focal brucellosis. Pak. J. Biol. Sci..

[21] Baud D, Greub G (2011). Intracellular bacteria and adverse pregnancy outcomes. Clin. Microbiol. Infect..

[22] Miao EA, Andersen-Nissen E, Warren SE, Aderem A (2007). TLR5 and Ipaf: Dual sensors of bacterial flagellin in the innate immune system. Semin. Immunopathol..

[23] Ting JP, Willingham SB, Bergstralh DT (2008). NLRs at the intersection of cell death and immunity. Nat. Rev. Immunol..

[24] Kersse K, Bertrand MJ, Lamkanfi M, Vandenabeele P (2011). NOD-like receptors and the innate immune system: Coping with danger, damage and death. Cytokine Growth Factor Rev..

[25] Das PM, Ramachandran K, Vanwert J, Ferdinand L, Gopisetty G, Reis IM, Singal R (2006). Methylation mediated silencing of TMS1/ASC gene in prostate cancer. Mol. Cancer.

[26] Rathinam VA (2010). The AIM2 inflammasome is essential for host defense against cytosolic bacteria and DNA viruses. Nat. Immunol..

[27] Gomes MT (2013). Critical role of ASC inflammasomes and bacterial type IV secretion system in caspase-1 activation and host innate resistance to *Brucella*
*abortus* infection. J. Immunol..

[28] Miraglia MC (2016). Glial cell–elicited activation of brain microvasculature in response to *Brucella*
*abortus* infection requires ASC inflammasome-dependent IL-1β production. J. Immunol..

[29] Marim FM, Franco M, Gomes M, Miraglia MC, Giambartolomei GH, Oliveira SC (2017). The role of NLRP3 and AIM2 in inflammasome activation during *Brucella*
*abortus* infection. Semin. Immunopathol..

[30] Karaca G (2019). The relationship between caspase-1 related inflammasome expression and serum inflammatory cytokine levels during acute brucellosis. North. Clin. Istanb..

[31] Winer J, Jung CK, Shackel I, Williams PM (1999). Development and validation of real-time quantitative reverse transcriptase–polymerase chain reaction for monitoring gene expression in cardiac myocytes in vitro. Anal. Biochem..

[32] Ariza J, Corredoira J, Pallares R, Viladrich PF, Rufi G, Pujol M, Gudiol F (1995). Characteristics of and risk factors for relapse of brucellosis in humans. Clin. Infect. Dis..

[33] Ögredici Ö (2010). Brucellosis reactivation after 28 years. Emerg. Infect. Dis..

[34] Campos PC, Gomes MT, Guimarães G, Costa Franco MM, Marim FM, Oliveira SC (2014). *Brucella*
*abortus* DNA is a major bacterial agonist to activate the host innate immune system. Microbes Infect..

[35] Pengbo C, Yali Z, Yuanzhi W, Shuxiang W, Huiqin W, Chuangfu C (2014). Preliminary study on the role of NLRP3 inflammasome in the infection of human macrophages THP-1 by Brucella bovis 2308 and RB51. J. Shihezi Univ. (Nat. Sci. Ed.).

[36] Tupik JD, Coutermarsh-Ott SL, Benton AH, King KA, Kiryluk HD, Caswell CC, Allen IC (2020). ASC-mediated inflammation and pyroptosis attenuates *Brucella*
*abortus* pathogenesis following the recognition of gDNA. Pathogens.

[37] Martinon F, Burns K, Tschopp J (2002). The inflammasome: A molecular platform triggering activation of inflammatory caspases and processing of proIL-beta. Mol. Cell.

[38] Rathinam VAK, Vanaja SK, Fitzgerald KA (2012). Regulation of inflammasome signaling. Nat. Immunol..

[39] Terra JK (2010). Cutting edge: resistance to Bacillus anthracis infection mediated by a lethal toxin sensitive allele of Nalp1b/Nlrp1b. J. Immunol..

[40] Pengbo, C. In *Preliminary study on the role of NLRP3 inflammasome in Brucella infection* (ed. Chuangfu, C.) (2014).

[41] Kayagaki N (2011). Non-canonical inflammasome activation targets caspase-11. Nature.

[42] Kerur N, Veettil MV, Sharma-Walia N, Bottero V, Sadagopan S, Otageri P, Chandran B (2011). IFI16 acts as a nuclear pathogen sensor to induce the inflammasome in response to Kaposi Sarcoma-associated herpesvirus infection. Cell Host Microbe.

[43] Silveira TN, Gomes MTR, Oliveira LS, Campos PC, Machado GG, Oliveira SC (2017). NLRP12 negatively regulates proinflammatory cytokine production and host defense against *Brucella*
*abortus*. Eur. J. Immunol..

