# Supplementary material for: Expression of NLRP3 and AIM2 inflammasome in Peripheral blood in Chinese patients with acute and chronic brucellosis
Source: Sci Rep. 2022 Sep 6;12:15123. doi: 10.1038/s41598-022-19398-9 (PMC9448728; doi:10.1038/s41598-022-19398-9)
Supplement: Supplementary file 1 — Supplementary Information. [file 41598_2022_19398_MOESM1_ESM.pdf]

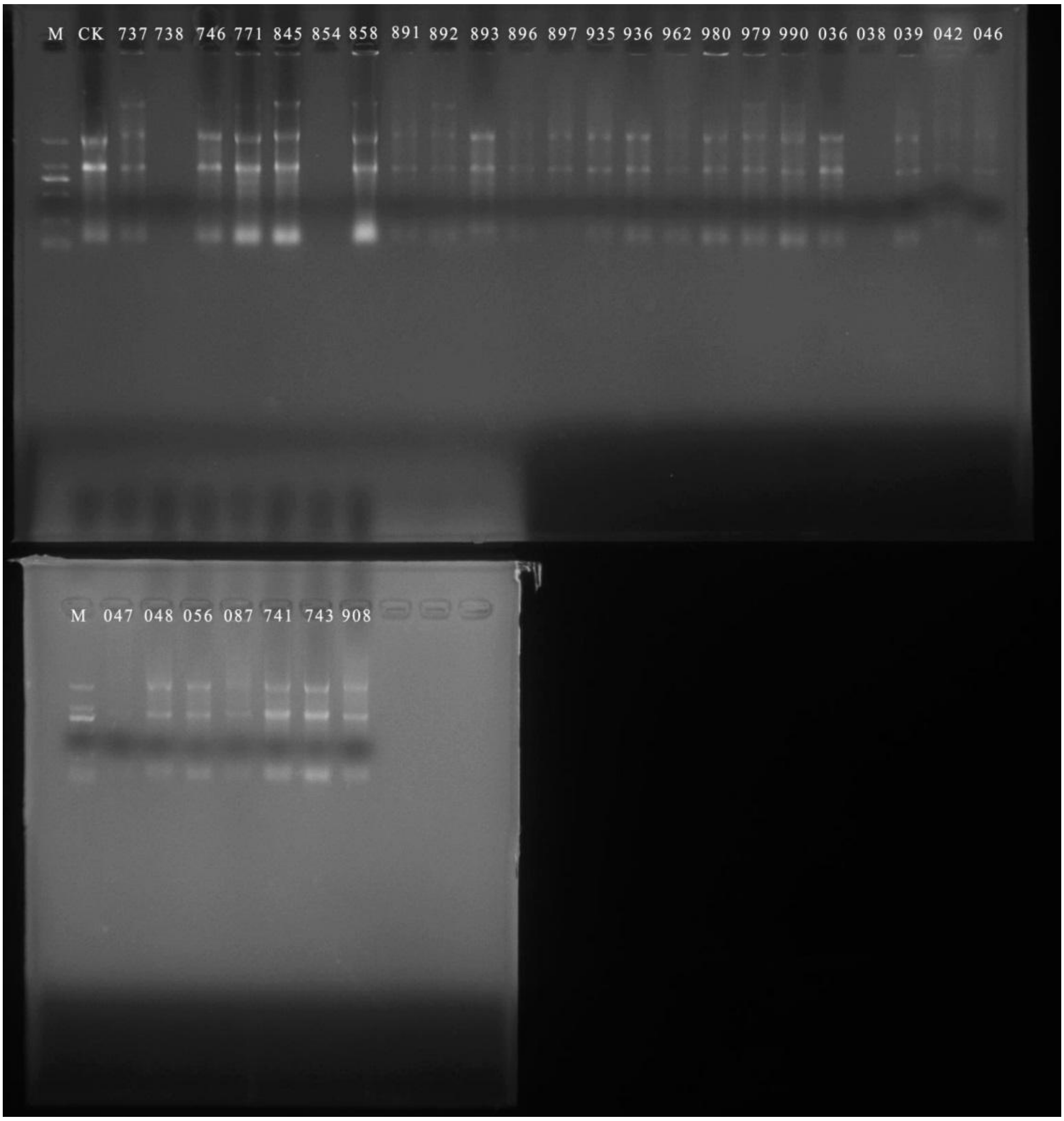
M CK 737 738 746 771 845 854 858 891 892 893 896 897 935 936 962 980 979 990 036 038 039 042 046
M 047 048 056 087 741 743 908

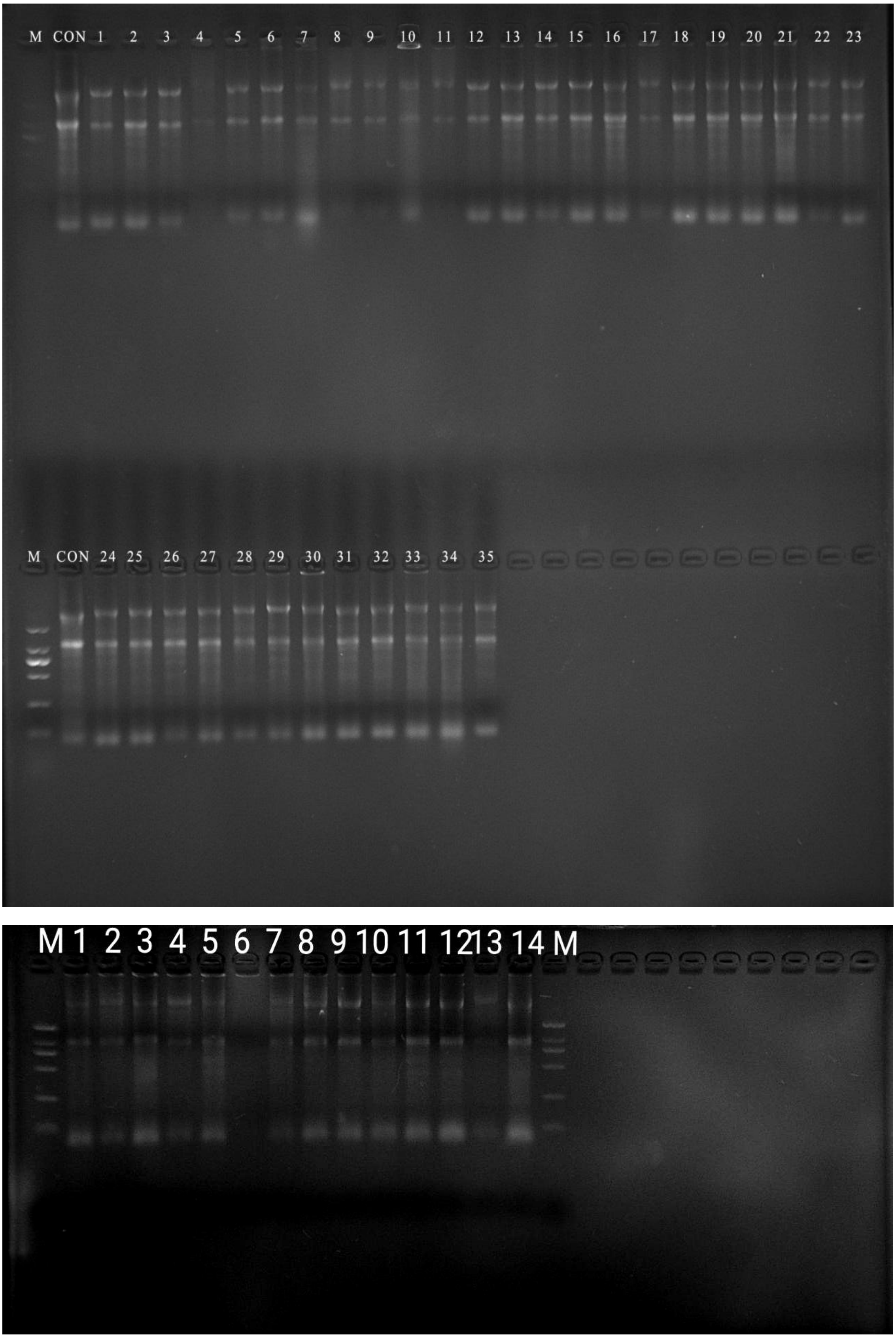

Figure S1 RNA integrity. Total RNA samples were extracted from peripheral blood using RNAiso Blood (TAKARA) according to the manufacturer’s instructions.The quality and quantity of RNA samples were evaluated by MaestroNano Spectrophotometer, and the

integrity was confirmed by electrophoresis (1.5% denaturing agarose gel electrophoresis).Only RNA samples that met the criteria were included in the study.

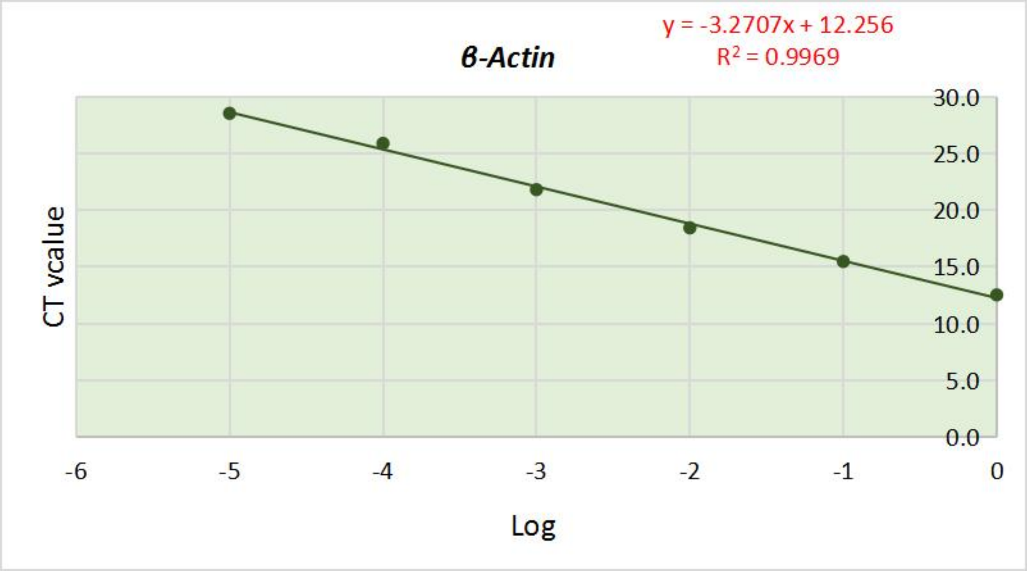

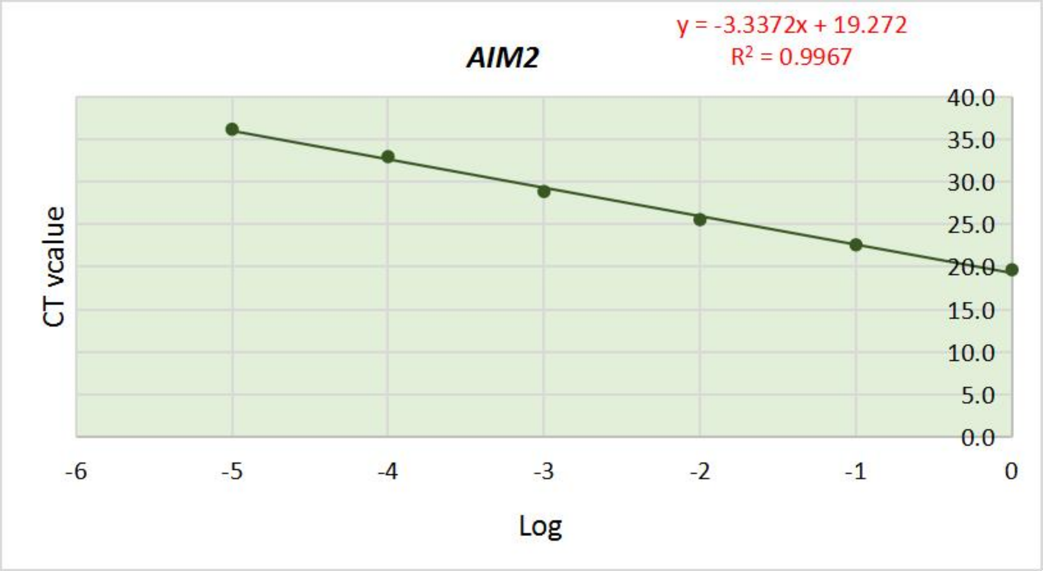

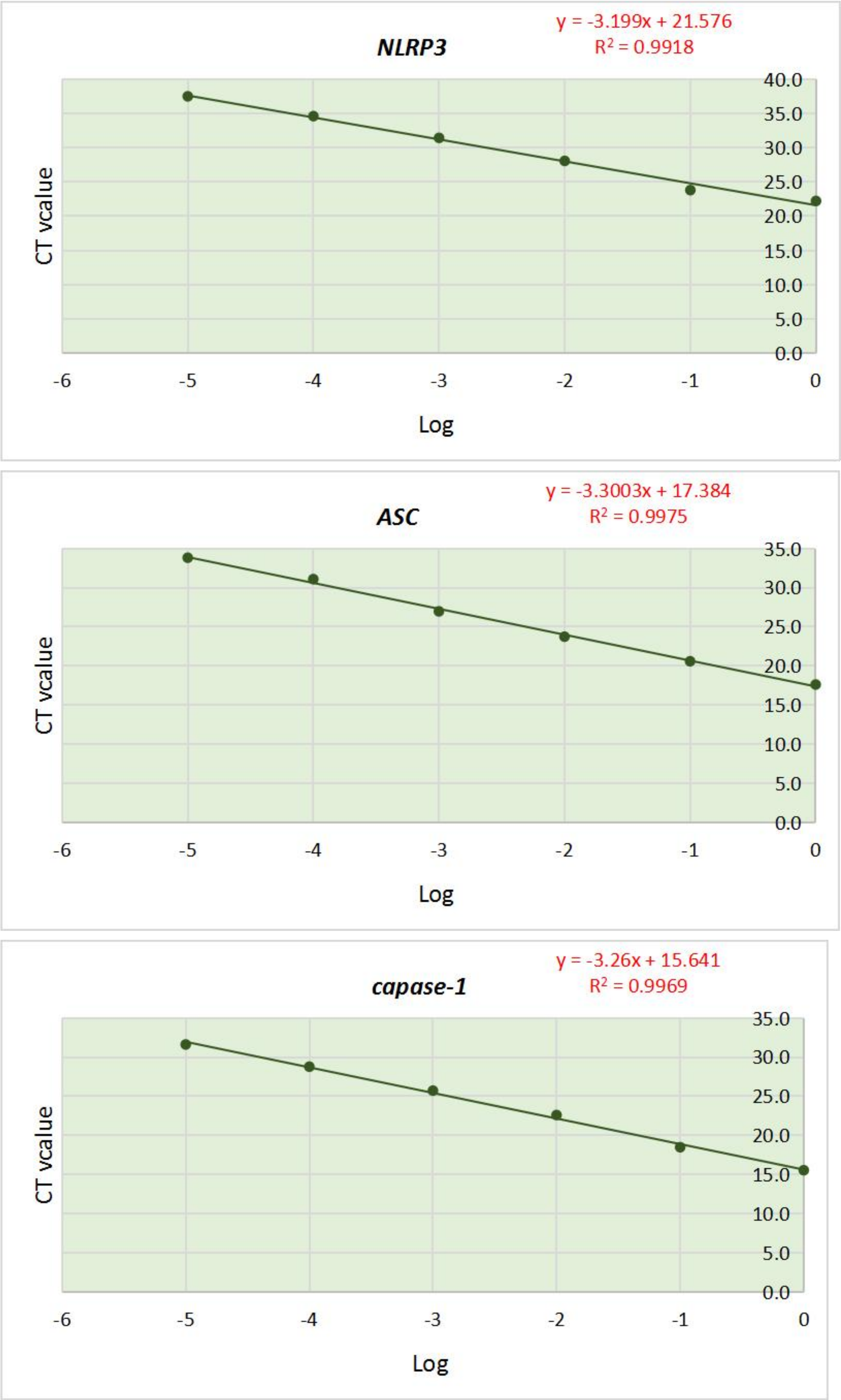

Figure S2 QPCR amplification efficiency standard curve.

TableS1 Baseline Supplementary Table for Case and Healthy Groups

Numbering	Enrollment	gender	age	RBPT	SAT	course of disease （months）	Cause of infection	antibiotics
25746	Acute	Male	65	++	1:100++	2	herdsmen, contact sheep	NO
25893	Acute	Male	54	++	1:100++++	1	herdsmen, contact sheep	NO
25935	Acute	female	46	++	1:100++	0.5	herdsmen, contact sheep	NO
26113	Acute	Male	32	++	1:100++	1	milk factory worker	NO
26118	Acute	Male	48	++	1:100++	2	milk factory worker	NO
26552	Acute	female	63	++	1:100++++	1.5	milk factory worker	NO
26587	Acute	Male	23	++	1:100++++	0.5	milk factory worker	NO
26932	Acute	Male	12	++	1:100++	0.5	Livestock company worker	NO
26953	Acute	Male	51	++	1:100++++	0.5	drink fresh milk	NO
27164	Acute	Male	23	++	1:100++	0.5	herdsmen, contact sheep	NO
27331	Acute	Male	36	++	1:100++	2	drink fresh milk	NO
27418	Acute	female	46	++	1:100++	0.5	drink fresh milk	NO
27456	Acute	Male	40	++	1:100++++	0.2	herdsmen, contact sheep	NO
27465	Acute	Male	44	++	1:100++++	0.2	herdsmen, contact sheep	NO
27513	Acute	Male	49	++	1:100++	0.5	milk factory worker	NO
27514	Acute	female	62	++	1:100++	1	drink fresh milk	NO
27603	Acute	Male	23	++	1:100++++	0.2	eating unsanitary lamb	NO
27703	Acute	female	36	++	1:100++++	0.2	herdsmen, contact sheep	NO
27814	Acute	Male	37	++	1:100++++	0.5	herdsmen, contact sheep	NO
26167	Acute	Male	23	++	1:100+++	0.2	eating unsanitary lamb	NO
25737	Chronic	female	34	++	1:100++	24	drink fresh milk	NO
25891	Chronic	female	54	++	1:100+++	48	drink fresh milk	NO
25892	Chronic	Male	39	++	1:50++	60	milk factory worker	NO
25896	Chronic	Male	45	++	1:100++	48	milk factory worker	NO
25897	Chronic	Male	63	++	1:100++	36	Livestock company worker	NO
26036	Chronic	female	44	++	1:100++	60	Livestock company worker	NO
26042	Chronic	Male	24	++	1:100++	48	Livestock company worker	NO
26586	Chronic	female	34	++	1:50++	18	drink fresh milk	NO
26588	Chronic	Male	31	++	1:100++++	48	herdsmen, contact sheep	NO
26935	Chronic	female	22	++	1:50++	48	herdsmen, contact sheep	NO
26944	Chronic	Male	36	++	1:50++	24	herdsmen, contact sheep	NO
26948	Chronic	female	39	++	1:100++	120	drink fresh milk	NO
27349	Chronic	Male	64	++	1:100++	48	drink fresh milk	NO
27481	Chronic	Male	51	++	1:100++++	24	Livestock company worker	NO
27620	Chronic	Male	57	++	1:100+++	24	herdsmen, contact sheep	NO
27822	Chronic	female	61	++	1:50++	12	herdsmen, contact sheep	NO
25962	Chronic	Male	57	++	1:50++	12	herdsmen, contact sheep	NO
25979	Chronic	female	32	++	1:50++	84	herdsmen, contact sheep	NO
25980	Chronic	Male	54	++	1:50++	12	eating unsanitary lamb	NO

25990	Chronic	Male	48	++	1:100+++	12	eating unsanitary lamb	NO
25858		female	26	-	-			NO
26846		Male	38	-	-			NO
26852		female	44	-	-			NO
26876		Male	54	-	-			NO
26879		Male	34	-	-			NO
26888		Male	23	-	-			NO
26890		female	56	-	-			NO
26940		Male	45	-	-			NO
26942		Male	43	-	-			NO
26945		female	36	-	-			NO
26946		female	51	-	-			NO
26947		female	34	-	-			NO
26949		Male	30	-	-			NO
26950		Male	41	-	-			NO
26955		Male	27	-	-			NO
26959		Male	26	-	-			NO
26961		female	35	-	-			NO
26962		female	26	-	-			NO
26964		Male	31	-	-			NO
26965		Male	60	-	-			NO

Table S2 QPCR amplification efficiency of each gene

**Gene**	**R^2**	**slope**	**Amplification efficiency E**
β-Actin	0. 9969	−3. 2707	102. 18%
capase-1	0. 9976	−3. 26	102. 65%
ASC	0. 9975	−3. 3003	100. 91%
AIM2	0. 9967	−3. 3372	99. 37%
NLRP3	0. 9918	−3. 199	105. 40%
